# Profile analysis and functional modeling identify circular RNAs in nonalcoholic fatty liver disease as regulators of hepatic lipid metabolism

**DOI:** 10.3389/fgene.2022.884037

**Published:** 2022-09-15

**Authors:** Yang Xie, Yi Cao, Can-Jie Guo, Xing-Ya Guo, Ya-Fang He, Qing-Yang Xu, Feng Shen, Qin Pan

**Affiliations:** ^1^ Department of Gastroenterology, School of Medicine, Xinhua Hospital, Shanghai Jiao Tong University, Shanghai, China; ^2^ Department of Pediatric Digestion and Nutrition, School of Medicine, Xinhua Hospital, Shanghai Jiao Tong University, Shanghai, China; ^3^ Department of Gastroenterology, School of Medicine, Renji Hospital, Shanghai Jiao Tong University, Shanghai, China; ^4^ Department of Gastroenterology, School of Medicine, Shanghai General Hospital, Shanghai Jiao Tong University, Shanghai, China; ^5^ Department of Pediatric Respiratory, School of Medicine, Xinhua Hospital, Shanghai Jiao Tong University, Shanghai, China; ^6^ Endoscopy Center, School of Medicine, Xinhua Hospital, Shanghai Jiao Tong University, Shanghai, China; ^7^ Research Center, Shanghai University of Medicine and Health Sciences Affiliated Zhoupu Hospital, Shanghai, China

**Keywords:** nonalcoholic fatty liver disease, circular RNA, microRNA, AMPK, signaling pathway

## Abstract

Nonalcoholic fatty liver disease (NAFLD) is the leading cause of chronic liver disease, associated with an outcome of hepatic fibrosis/cirrhosis and hepatocellular carcinoma. However, limited exploration of the underlying mechanisms hinders its prevention and treatment. To investigate the mechanisms of epigenetic regulation in NAFLD, the expression profile of circular RNA (circRNA) of rodents in which NAFLD was induced by a high-fat, high-cholesterol (HFHC) diet was studied. Modeling of the circRNA-microRNA (miRNA) -mRNA regulatory network revealed the functional characteristics of NAFLD-specific circRNAs. The targets and effects in the liver of such NAFLD-specific circRNAs were further assessed. Our results uncovered that the downregulation of 28 annotated circRNAs characterizes HFHC diet-induced NAFLD. Among the downregulated circRNAs, long intergenic non-protein coding RNA, P53 induced transcript (LNCPINT) -derived circRNAs (circ_0001452, circ_0001453, and circ_0001454) targeted both miR-466i-3p and miR-669c-3p. Their deficiency in NAFLD abrogated the circRNA-based inhibitory effect on both miRNAs, which further inactivated the AMPK signaling pathway *via* AMPK-α1 suppression. Inhibition of the AMPK signaling pathway promotes hepatic steatosis, depending on the transcriptional and translational upregulation of lipogenic genes, such as those encoding sterol regulatory element-binding protein 1 (SREBP1) and fatty acid synthase (FASN) in hepatocytes. The levels of LNCPINT-derived circRNAs displayed a negative association with hepatic triglyceride (TG) concentration. These findings suggest that loss of LNCPINT-derived circRNAs may underlie NAFLD *via* miR-466i-3p- and miR-669c-3p-dependent inactivation of the AMPK signaling pathway.

## Introduction

Nonalcoholic fatty liver disease (NAFLD) is a pathological disorder of hepatic steatosis in association with multiple components of metabolic syndrome (*e.g*., obesity, type 2 diabetes, hyperlipidemia) (Younossi et al., 2016). Being attributed to the high-fat diet, sedentary behavior, and related obesity/overweight, the past decades have witnessed a remarkable increase in the prevalence of NAFLD, which has reached 13.48% of the population in Africa, 25% in Asia, 23.71% in Europe, 31.79% in the Middle East, 24.13% in North America, and 30.45% in South America (Younossi et al., 2016; Fan et al., 2017). NAFLD is a spectrum disease, encompassing several conditions such as nonalcoholic fatty liver, nonalcoholic steatohepatitis, liver fibrosis/cirrhosis, and even hepatocellular carcinoma (Fan et al., 2017). Numerous interacting complicated mechanisms, such as the occurrence of risk gene factors, epigenetic alterations, and gut dysbiosis, underlie the occurrence of NAFLD. The lack of knowledge of such pathogenic events has limited the development of effective therapies other than lifestyle interventions (Caligiuri et al., 2016).

Various miRNAs have been recognized as key regulators of hepatic steatosis based on their complementation to the 3′ untranslated region (3’ UTR) of target mRNAs (Bartel, 2009). For example, miR-30a-3p and miR-3666 protect hepatocytes from steatosis by targeting peroxisome proliferator-activated receptor (PPAR)-α and PPAR-*γ*, respectively (Wang et al., 2020a; Mittal et al., 2020). miR-29a (Mattis et al., 2015), miR-185 (Wang et al., 2014), and miR-192-3p (Wang et al., 2020b) also downregulate the expression of lipogenic genes, including those encoding the glucocorticoid receptor, FASN, 3-hydroxy-3-methylglutaryl-CoA reductase (HMGCR), and SREBP-1c/2, to inhibit hepatic lipogenesis. In contrast, the inhibitory effects of miR-291b-3p and miR-188-3p on AMPK-α1 and antioxidant enzyme glutathione peroxidase 4, respectively, promote hepatocyte steatosis (Meng et al., 2016; Yang et al., 2020). Similarly, miR-199a-5p contributes to impaired *ß*-oxidation of fatty acids and the accumulation of excessive lipid deposits in the liver (Li et al., 2014).

CircRNAs, a type of non-coding RNA, have recently been shown to sponge miRNAs by binding to miRNA response elements (MREs) (Hansen et al., 2013). The pathophysiological regulation of glycolipid metabolism by circRNAs relies on the sponging-based inactivation of the target miRNAs. Insulin resistance, which is considered the “first hit” in NAFLD, has been attributed to the sponging of miR-192-5p and miR-145 by circHIPK3 and circANKRD36, respectively (Cai et al., 2020; Lu et al., 2021). Differential expression of circACC1 controls lipid storage by modulating fatty acid *ß*-oxidation and glycolysis (Li et al., 2019a). Upon high-fat stimulation, circRNA_0046367 loss in HepG2 cells reactivates the miR-34a/PPARα axis, inducing hepatocellular steatosis (Guo et al., 2017). Thus, circRNA/miRNA interactions establish a novel yet critical layer of epigenetic regulation in hepatic steatosis. However, these interactions and their roles are still poorly understood.

In this study, we investigated the profile of hepatic circRNAs in rodents in which NAFLD was induced by a high-fat, high-cholesterol diet. The circRNA-miRNA-mRNA regulatory network was then established based on target prediction at both the miRNA and mRNA levels. Pathway analysis and Kyoto Encyclopedia of Genes and Genomes (KEGG) annotation further revealed the functional characteristics of the elements of the circRNA-miRNA-mRNA network. To investigate the mechanism of action of NAFLD-specific circRNAs, their expressive alterations and related effects on target miRNAs were quantified in the liver of NAFLD mice. Moreover, downstream genes and signaling pathways underlying the effect of circRNAs were subjected to bioinformatics, transcriptional and translational identification. *In situ* labeling was used to test the concurrence and colocalization of hepatic steatosis and the expression of lipogenic genes.

## Materials and methods

### Rodent model of NAFLD

Adult male specific pathogen free (SPF) c57BL/6 mice (Jihui Laboratory Animal Care Co., Shanghai, China) were randomized using the lottery method into the normal control (NC) group (*n* = 6), which was fed a normal diet, and the NAFLD group (*n* = 6), which was fed an HFHC diet (2% cholesterol, 10% lard, and 88% normal diet). The sample size was estimated by the prospective difference in circRNAs between the NC and NAFLD groups using Gpower v3.1.9.7 software (Faul et al., 2009). The mice were measured for body weight weekly and raised in specific pathogen free (SPF) environment, with a 12 h light/12 h dark cycle, 24 ± 2°C ambient temperature, 50 ± 5% humidity and free access to water and pelleted chow. The HFHC diet was administrated for 16 weeks to establish a rodent model of NAFLD (Liu et al., 2020). And liver samples were collected at the end of the experiments and measured for liver weight. The liver index of the mice was calculated as follow: liver index = liver weight/bodyweight. All animal experiments were conducted under the Guideline for the Care and Use of Laboratory Animals (1996), with the approval of the ethical committee of Xinhua hospital.

### Pathological assessment

Liver samples were collected from each mouse and embedded with paraffin or optimal cutting temperature (OCT) compound for pathological evaluation after the 16-weeks treatment. Frozen sections of OCT-embedded liver samples were stained with Oil Red O to assess the accumulation of neutral lipids (Mehlem et al., 2013). Hematoxylin-eosin (HE) staining of paraffin sections was used to evaluate the NAFLD-specific characteristics according to the NAS method (Kleiner et al., 2005). The steatosis was ranked by the area of hepatic steatosis according to S0 < 5%, S1>5%–33%, S2>33%–66%, S3>66%. The pathological alterations in each sample were assessed by two pathologists in a blinded manner.

### Analysis of triglyceride concentration

Liver tissue was ground into a homogenate and centrifuged for the supernatant. The total protein concentration of the supernatant was determined using a BCA protein concentration determination kit G2026 (Servicebio, Wuhan, China). The BCA working solution was prepared by Bicinchonic acid and copper sulfate solution in 50: 1 ratio of volume. The supernatant (2 μl) was diluted in 18 μl phosphate buffer saline (PBS) buffer, mixed with 200 μl working solution, and incubated at 37°C for half an hour. After the incubation, the optical density (OD) was measured using BioTek Epoch Microplate Spectrophotometer (Agilent technologies, Santa Clara, CA, United States) at 562 nm wave length. The standard curve was calculated by the protein concentrations of 0, 25, 50, 100, 200, 300, 400, 500 μg/ml against the optical density obtained under the same condition. The concentration of total protein was calculated using the optical density according to the standard curve. TG contents in the liver of mice in both NC and NAFLD groups were measured using the TG Determination Kit (GPO-PAP method) (C061, Huili, Changchun, China) against total hepatic protein contents (Zhang et al., 2015). The 250 μl GPO-PAP working solution was mixed with 2.5 μl PBS, standard glycerol (1.7 mmol/L), or supernatant, respectively. The mixture was then subjected to automatic biochemistry analyzer (Chemray 800, Rayto, Shenzhen, China) and analyzed the OD at 300 s and 510 nm wave length. The TG concentration was determined using colorimetric method with standard glycerol after zero set with PBS.

### circRNA sequencing

Total RNA was extracted from the liver of mice in NC (*n* = 3) and NAFLD (*n* = 3) groups using RNAiso Plus reagent (Takara Bio, Shiga, Japan). The concentration and purity of hepatic RNA were evaluated using the NanoDrop ND-100 (Thermo Fisher Scientific, Waltham, MA, United States) and its integrity was assessed using the Agilent Bioanalyzer 2,100 (Agilent technologies). After excluding ribosomal and linear RNA, circRNA samples were subjected to cDNA synthesis, end repair, 3′ end adenylation, and adapter ligation. The cDNA library was then constructed, and its concentration and size were assessed using the Qubit^®^ 2.0 Fluorometer (Thermo Fisher Scientific, Waltham, MA, United States) and Agilent Bioanalyzer 2,100, respectively. Paired end sequencing was performed using Illumina HiSeq 2,500 (Illumina, San Diego, CA, United States) following the manufacturer’s instruction. Clean reads were further filtered using Seqtk (https://github.com/lh3/seqtk) and mapped to reference genome GRCm38 using the BWA-MEM alignment algorithm (http://bio-bwa.sourceforge.net/). Finally, the hepatic circRNA profile was obtained from clean reads using the CIRI algorithm (Gao et al., 2015) against circBase (http://circrna.org/) (Glažar et al., 2014). After normalizing data using spliced reads per billion mapping (SRPBM) (Jeck et al., 2013), differentially expressed circRNAs in the NAFLD group were obtained using the edgeR R package (Robinson et al., 2010) based on the criteria of fold-change ≥ 2 and *p* value < 0.05.

### Bioinformatic modeling

First, circRNA sets identified from the liver of NC and NAFLD groups were intersected to shed light on the scale and difference of their expression profile. The sequences of these circRNAs were mapped to the reference genome GRCm38 by CIRI software to uncover their chromosomal and genic distribution. The differentially expressed circRNAs were further subjected to unsupervised hierarchical clustering using TreeView and depicted using a volcano plot.

Second, the interactions between differentially expressed circRNAs and target miRNAs were investigated by analyzing the base complementation between the MREs of circRNA and the seed sequence of miRNA using the miRanda algorithm (Version:3.3a, https://omictools.com/miranda-tool) (Enright et al., 2003). The key circRNAs involved in miRNA regulation were filtered using the following parameters: score >180, energy < −30, and degree of circRNA-miRNA network ≥2. Moreover, downstream mRNAs of the circRNA-targeted miRNAs were investigated using integrated analysis of miRNA databases (miRDB (Chen and Wang, 2020), Targetscan (Agarwal et al., 2015), miRanda (Enright et al., 2003), miRWalk (Dweep and Gretz, 2015), miRMap (Vejnar and Zdobnov, 2012), miRNAMap (Hsu et al., 2008), RNA22 (Miranda et al., 2006)). Those mRNAs found in ≥6 databases, together with the miRDB score >80, were considered to be crucial in miRNA-based gene regulation (Chen and Wang, 2020). The circRNA-miRNA-mRNA regulatory network was finally established using Cytoscape v 3.8.2 (https://cytoscape.org/) (Shannon et al., 2003).

Third, target miRNA sets of circRNAs were intersected to reveal the characteristics of the circRNA-miRNA-mRNA regulatory network. To highlight the pathophysiological effects of the intersecting miRNAs, their target mRNA sets were subjected to KEGG enrichment analysis using the clusterProfiler R package (Yu et al., 2012; Kanehisa et al., 2021). Top-ranking signaling pathways with significant *p*-values were visualized using the ggplot2 3.3.0 R package (https://ggplot2.tidyverse.org/). The protein-protein interaction network of the key signaling pathway was constructed using the STRING database (https://cn.string-db.org/) (Szklarczyk et al., 2021) and modified using Cytoscape v3.8.2.

### Quantitative reverse transcription polymerase chain reaction (RT-QPCR)

The circRNAs and mRNAs of each liver sample were extracted and subjected to reverse transcription with random primers and oligo dT primers using PrimeScript™ RT Reagent Kit (Takara Bio, Shiga, Japan) according to the manufacturer’s instructions, while the RT of miRNAs and U6 was performed with specific stem-loop RT primers and U6 reverse primers using the same method respectively. Quantitative PCR was performed on circRNAs, miRNAs, and mRNAs using Hieff UNICON^®^ qPCR SYBR^®^ Green Master Mix (Low Rox) (Yeasen Biotechnology, Shanghai, China) on QuantStudio 3 (Thermo Fisher Scientific, Waltham, MA, United States) with three replications. Relative expression levels were normalized to GAPDH (circRNAs and mRNAs) or U6 (miRNAs) housekeeping genes using the 2^−ΔΔct^ method. The divergent primers (circRNAs), convergent primers (miRNAs and mRNAs), and specific stem-loop primers used in the experiments are shown in Table 1.

**TABLE 1:** Specific primers for RT-QPCR.

Name (ID)	Type	Sequence (5′-3′)	Product size (Bp)
Circ_0001452	Forward	TAG​CAG​CAA​CCT​GGT​CTC​TTT	119
	Reverse	ATC​AGC​AAG​GCA​GAG​AGG​TG	
Circ_0001453	Forward	GCA​ACC​TGG​TCT​CTT​TTC​ACG	104
	Reverse	CCC​GAA​AGT​GAG​GCA​AGG​AC	
Circ_0001454	Forward	TGA​CTC​TGG​GCT​CTC​AGG​AA	216
	Reverse	ATC​AGC​AAG​GCA​GAG​AGG​TG	
Circ_0000503	Forward	CAG​TCG​TGG​AAT​CTG​TGG​GT	242
	Reverse	TCA​TCC​AGC​TCT​GCT​CCA​TC	
MiR-466i-3p (MI0006282)	Stem-loop	GTC​GTA​TCC​AGT​GCA​GGG​TCC​GAG	66
		GTA​TTC​GCA​CTG​GAT​ACG​ACT​AGT​GT	
	Forward	GGC​ATA​CAC​ACA​CAC​ATA​CAC​AC	60
	Reverse	AGT​GCA​GGG​TCC​GAG​GTA​TT	
MiR-669c-3p (MI0004673)	Stem-loop	GTC​GTA​TCC​AGT​GCA​GGG​TCC​GAG	66
		GTA​TTC​GCA​CTG​GAT​ACG​ACT​TTA​CT	
	Forward	CGC​TAC​ACA​CAC​ACA​CAC​AAG​TAA​A	60
	Reverse	AGT​GCA​GGG​TCC​GAG​GTA​TT	
AMPK-α1 (105787)	Forward	GCC​TTG​AAA​GAA​GTG​TGT​GAG​AAG​TTC	85
	Reverse	GTG​GGT​CCT​GGT​GGT​TTC​TGT​TG	
SREBP1 (20787)	Forward	GCT​ACC​GGT​CTT​CTA​TCA​ATG​A	91
	Reverse	CGC​AAG​ACA​GCA​GAT​TTA​TTC​A	
FASN (14104)	Forward	TAA​AGC​ATG​ACC​TCG​TGA​TGA​A	230
	Reverse	GAA​GTT​CAG​TGA​GGC​GTA​GTA​G	
GAPDH (14433)	Forward	GGT​TGT​CTC​CTG​CGA​CTT​CA	183
	Reverse	TGG​TCC​AGG​GTT​TCT​TAC​TCC	
U6 (19862)	Forward	CTC​GCT​TCG​GCA​GCA​CAT​ATA​C	82
	Reverse	ATT​TGC​GTG​TCA​TCC​TTG​CG	

AMPK-α1, Adenosine 5’-monophosphate-activated protein kinase α1; FASN, fatty acid synthase; GAPDH, glyceraldehyde-3-phosphate dehydrogenase; SREBP1, sterol regulatory element-binding protein 1.

### Western blot

Protein samples were harvested from mouse liver using RIPA lysis buffer and quantified by BCA protein concentration determination kit G2026 (Servicebio) with the same method. Total protein extracts were incubated with loading buffer at 100°C for 10 min and subjected to electrophoresis on 8% sodium dodecyl sulfate-polyacrylamide gel electrophoresis. Subsequently, proteins were transferred onto polyvinylidene difluoride membranes. The membranes were incubated with protein-free rapid blocking buffer for 20 min, and subsequently, with primary antibodies against *a*-tubulin (1:1,000, 66031, Proteintech, Rosemont, IL, United States), AMPK-α1 (1.5:1,000, AF6422, Affinity, Liyang, China), SREBP1 (1.5:1,000, AF6283, Affinity), and FASN (1:1,000, 10624, Proteintech) overnight at 4 °C, and with horseradish peroxidase (HRP) -conjugated secondary antibodies at room temperature for 1 h. Protein bands were visualized by chemiluminescence using AI680 (Cytiva, Marlborough, MA, United States) and assessed using ImageJ software (https://imagej.nih.gov/ij/) with integrated density/area.

### Immunohistochemical staining

The paraffin sections of liver samples were exposed to deparaffinization in xylene twice and ethanol three times sequentially for 5 min each time, rehydration with PBS, and antigen retrieval in boiled citrate buffer for 3 min. Then they were successively incubated with 3% hydrogen peroxide buffer in dark for 25 min, 3% bovine serum albumin for 30 min, and primary antibodies against AMPK-α1 (AF6422, 1:50, Affinity), SREBP1 (AF6283, 1:50, Affinity), and FASN (1:100, 10624, Proteintech) overnight at 4 °C in humidor. After reaction with HRP conjugated secondary antibody for 1 h, immunohistochemistry signals were visualized using the DAB method and assessed using ImageJ software with integrated density/area. The sections were washed in PBS 5 min for three times between every step mentioned above.

### Statistical analysis

The numerical variables of this study were presented as mean ± standard error (SEM). Kolmogorov-Smirnov test and F-test were applied for assessing the normality and homogeneity of variance of the data, respectively. To investigate the difference between NC and NAFLD groups, data following normal distribution were analyzed using Student’s t-test (homogenous variance) or Welch’s *t*-test (inhomogeneous variance). Data following a non-normal distribution were analyzed using the Mann-Whitney test. In addition, a two-way repeated-measures ANOVA was performed to assess the effect of diet on bodyweight. SPSS version 19.0 (SPSS Inc., Chicago, IL, United States) was used for the statistical analyses. Two-side significance threshold was set at *p* < 0.05.

## Results

### HFHC diet induced rodent NAFLD

**FIGURE 1:**
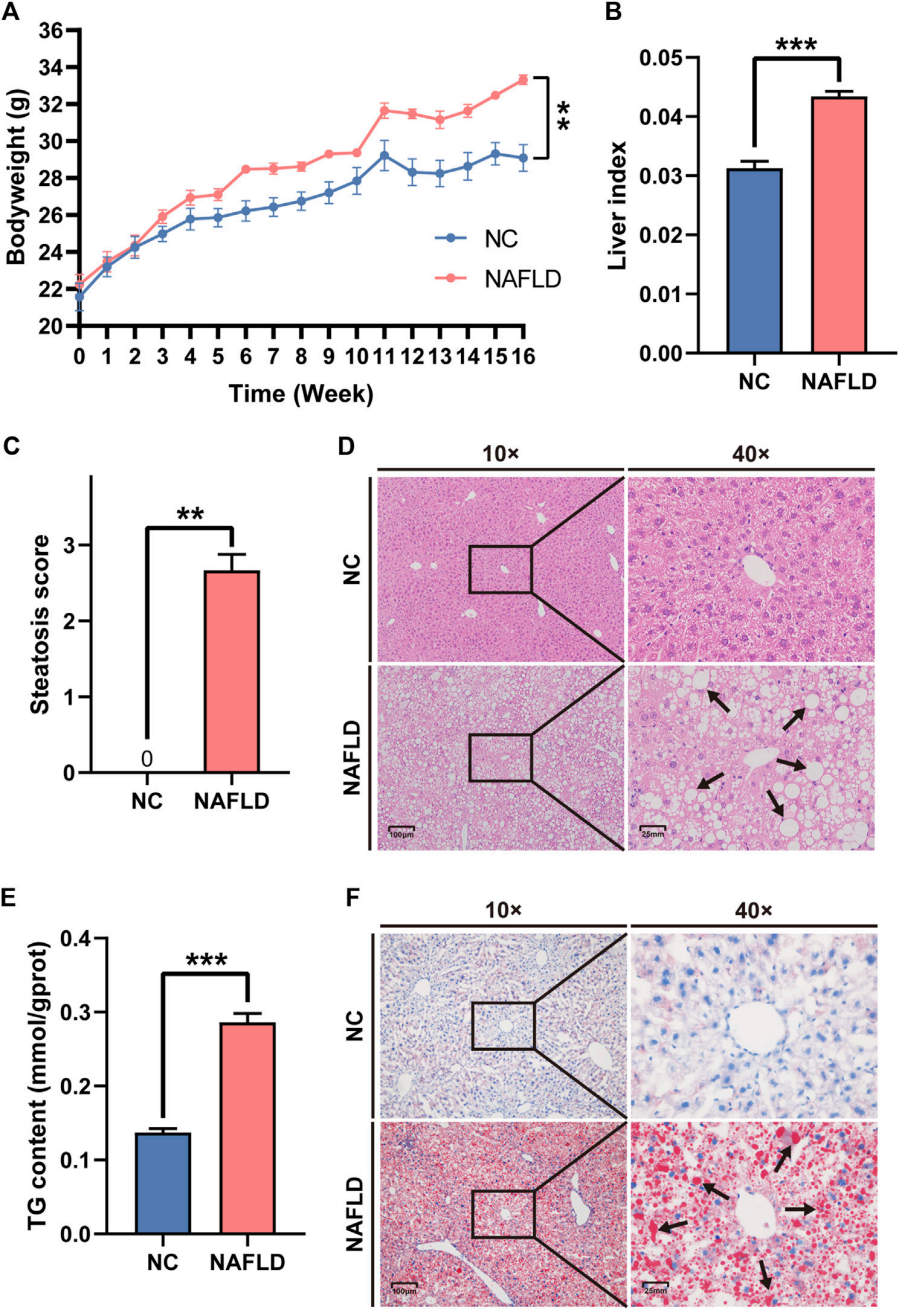
High-fat, high-cholesterol (HFHC) diet establishes rodent nonalcoholic fatty liver disease (NAFLD). **(A,B)** HFHC diet increased body weight **(A)** and liver weight **(B)** in the NAFLD group as compared to those in the NC group. **(C,E)** The NAFLD group demonstrated much higher steatosis score **(C)** and hepatic triglyceride (TG) concentration **(E)** than those in the NC group. **(D,F)** Both HE **(D)** and oil red O staining **(F)** exhibited the hepatocytes steatosis in the NAFLD instead of the NC group. Arrows reflect the lipid droplets in the cytoplasm of hepatocytes. The data was presented as mean ± SEM. **, *p*＜0.01; ***, *p* < 0.001.

Mice that were fed an HFHC diet for 16 weeks presented a bodyweight significantly higher than that of mice in the NC group fed a normal diet (*p* < 0.01, Figure 1A). The liver index in the HFHC diet group was much higher than that in the NC group (*p* < 0.001, Figure 1B). The pathological assessment showed that all mice in the NC group were graded S0 according to the steatosis score, while those in the NAFLD group were graded ≥ S1, which also indicated that hepatic steatosis was induced by the HFHC diet (Mann Whitney test: NC *vs*. NAFLD, 0 *vs*. 2.667 ± 0.211, *p* = 0.0022, Figure 1C). Additionally, mice fed an HFHC diet presented a significant upregulation of hepatic TG concentration compared to mice fed a normal diet (*p* < 0.001, Figure 1E). Moreover, HE and Oil Red O staining highlighted widespread, hepatocyte-enriched lipid droplets in mice fed an HFHC diet (Figures 1D,F), indicating the presence of hepatocyte steatosis, and hence, the successful establishment of the animal model of NAFLD.

### NAFLD-characteristic dysregulation of circRNAs

**FIGURE 2:**
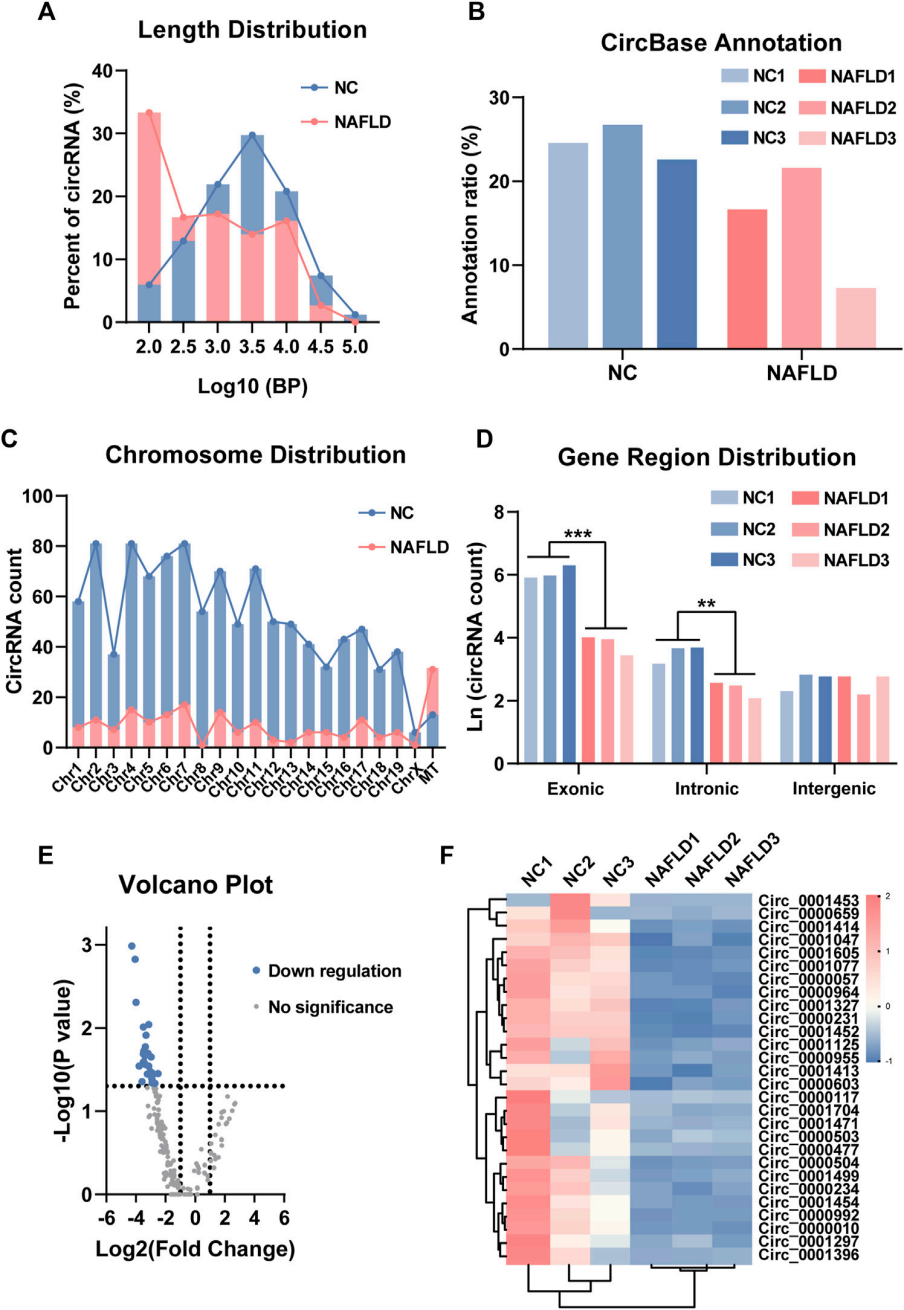
Circular RNA (circRNA) sequencing uncovers its profile in NAFLD. **(A)** The sequence length of circRNAs in the NC and NAFLD groups. **(B)** Annotation ratio reflected the percentage of circRNAs with circBase annotation. **(C)** circRNA count showed the chromosomal distribution of circRNAs. **(D)** The natural logarithm of circRNA count revealed their exonic, intronic, and intergenic derivation. **(E)** The volcano plot exhibited differentially expressed circRNAs with circBase annotation between the NC and NAFLD groups. **(F)** Heat map of 28 down-regulated circRNAs in the NAFLD group. The data was presented as mean ± SEM. **, *p* < 0.01; ***, *p* < 0.001.

The sequence length, annotation, chromosomal location, and gene region distribution of the liver circRNAs in the NC and NAFLD groups were investigated. Interestingly, the sequence length of circRNAs in the NC group and NAFLD group showed normal distribution and power-law distribution, respectively (Figure 2A). The annotation ratio of circRNAs was similar in both groups (Figure 2B). There were more circRNAs encoded by mitochondrial DNA than in chromosome 1–19 and chromosome X in the NAFLD group than in the NC group (Figure 2C). In addition, the natural logarithm of circRNA count revealed that they derived from a much more reduced number of exons and introns than those of the NC group (*p* < 0.01–0.001, Figure 2D).

Based on the fold-change and *p*-value of their expressive difference, 28 annotated circRNAs were found to be differentially expressed between the NC and NAFLD groups (Figures 2E,F). These NAFLD-related circRNAs were significantly downregulated in the NAFLD compared to the NC group (Figures 2E,F). Thus, both the number and expression level of circRNAs were reduced by HFHC diet-induced rodent NAFLD.

### *LNCPINT*-derived circRNAs affect the AMPK signaling pathway *via* miR-466i-3p and miR-669c-3p

**FIGURE 3:**
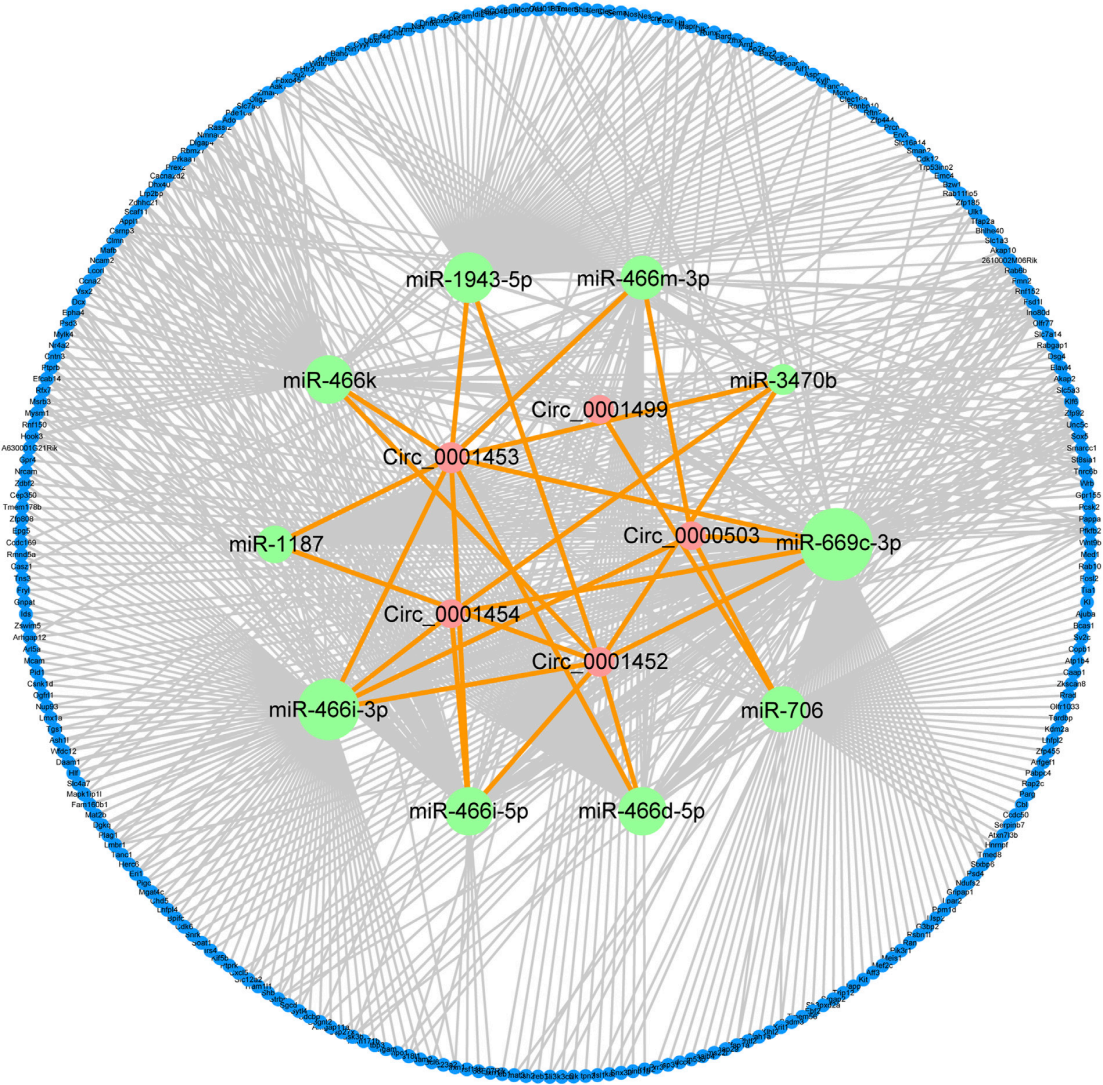
circRNA-miRNA-mRNA regulatory network characterizes the effects of NAFLD-related circRNAs. miRanda algorithm identified five key circRNAs (circ_0001452, circ_0001453, circ_0001454, circ_0000503, circ_0001499) with differential expression and their targeting of 10 key miRNAs. Database screening and miRDB scoring uncovered a group of 282 important mRNAs downstream of these key miRNAs. The integration of circRNA-miRNA and miRNA-mRNA interactions characterized the regulatory effects of circRNAs on NAFLD. Green, pink, and blue circles reflect circRNAs, miRNAs, and mRNAs, respectively. Orange and grey lines reflect the circRNA-miRNA and miRNA-mRNA interactions, respectively.

To gain insight into the roles of NAFLD-related circRNAs, their sponging ability was assessed using the miRanda algorithm, including the criteria of the score, energy, and degree of circRNA-miRNA network. Databases screening and miRDB scoring further revealed crucial mRNAs downstream of circRNA-regulated miRNAs. The circRNA-miRNA and miRNA-mRNA interactions formed a circRNA-miRNA-mRNA regulatory network comprised by five circRNAs (circ_0001452, circ_0001453, circ_0001454, circ_0000503, circ_0001499), 10 miRNAs (miR-1943-5p, miR-466m-3p, miR-3470b, miR-669c-3p, miR-706, miR-466d-5p, miR-466i-5p, miR-466i-3p, miR-1187, miR-466k), and 282 mRNAs (Figure 3).

**FIGURE 4:**
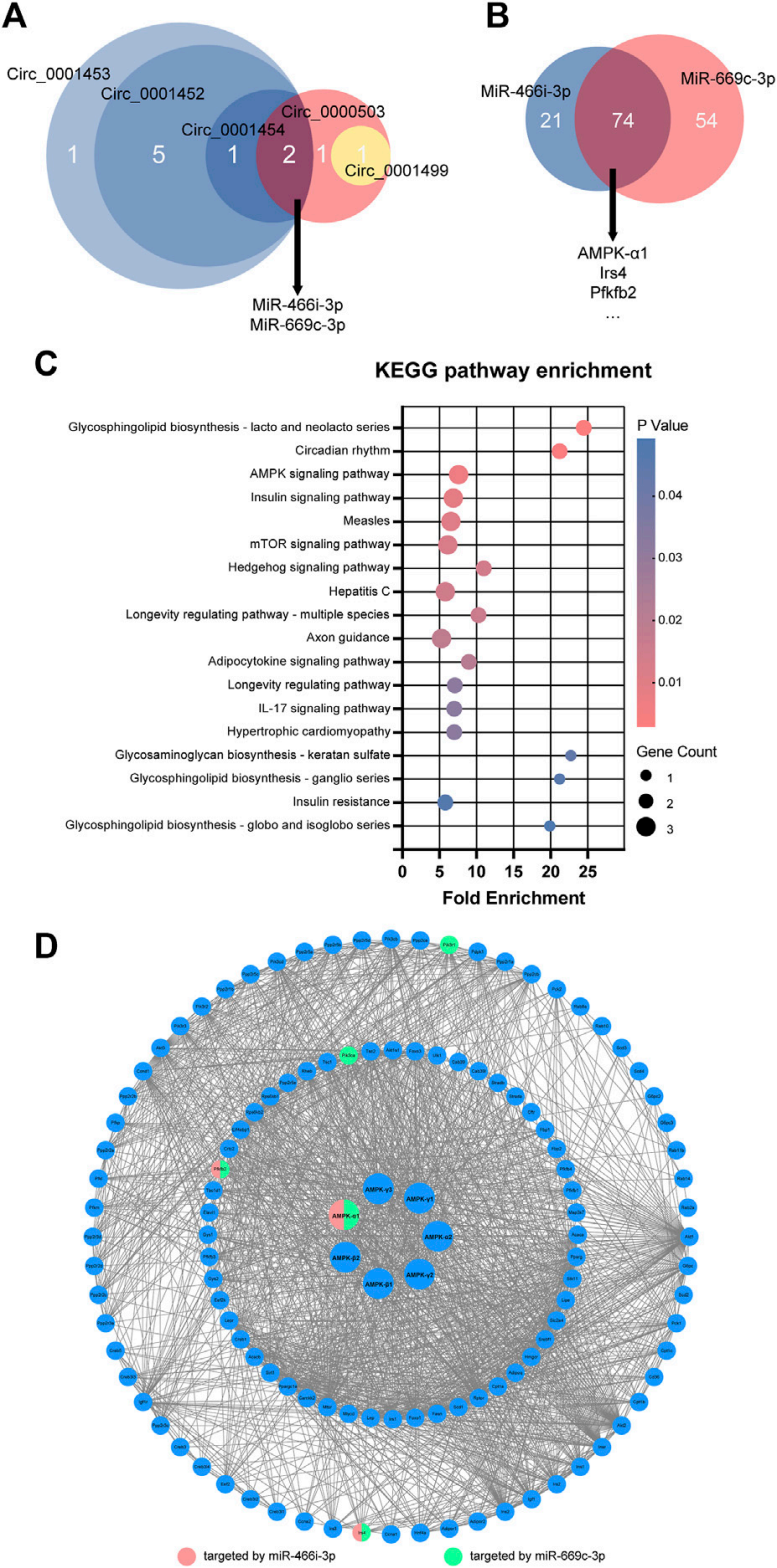
LNCPINT-derived circRNAs affected AMPK signaling pathway *via* miR-466i-3p- and miR-669c-3p-based AMPK regulation. **(A)** Target-miRNAs of five key circRNAs were intersected to show their focus on miR-466i-3p and miR-669c-3p. Most of these miR-466i-3p- and miR-669c-3p-targeting circRNAs (circ_0001452, circ_0001453, circ_0001454) shared the exonic derivation of LNCPINT. **(B)** A total of 74 mRNAs (*e.g*., AMPK-α1, Pfkfb2, and Irs4). reflected the common targets of miR-466i-3p and miR-669c-3p. **(C)** KEGG pathway analysis displayed significant enrichment of these common target mRNAs in the AMPK and other signaling pathways associated with glycolipid metabolism. **(D)** Both miR-466i-3p and miR-669c-3p exerted a regulatory effect on the AMPK signaling pathway via AMPK-α1.

Most of the key circRNAs (4/5) of the circRNA-miRNA-mRNA regulatory network shared the target miRNAs miR-466i-3p and miR-669c-3p (Figure 4A). circ_0001452, circ_0001453, and circ_0001454 were derived from *LNCPINT* (Gene ID: 232685). Additionally, miR-466i-3p and miR-669c-3p shared 77.84% (74/95) and 57.81% (74/128) of the target mRNAs, respectively (Figure 4B). Members of the AMPK signaling pathway (such as AMPK-α1, Pfkfb2, and Irs4) featured these miRNA targets (Figure 4B). KEGG pathway analysis further revealed the signaling pathways with top-ranking fold enrichment and *p* < 0.05, which included signaling pathways associated with the glycolipid metabolism, such as the AMPK, insulin, mTOR, and insulin resistance signaling pathways (Figure 4C). The gene encoding AMPK-α1 was the target of both miR-466i-3p and miR-669c-3p. Additionally, AMPK-α1 was a central hub in the protein-protein interaction network of the AMPK signaling pathway (Figure 4D). Therefore, *LNCPINT*-derived circRNAs were suggested to play an important role in NAFLD through miR-466i-3p- and miR-669c-3p-dependent regulation of the AMPK signaling pathway.

### Loss of *LNCPINT*-derived circRNAs activated miR-466i-3p and miR-669c-3p

**FIGURE 5:**
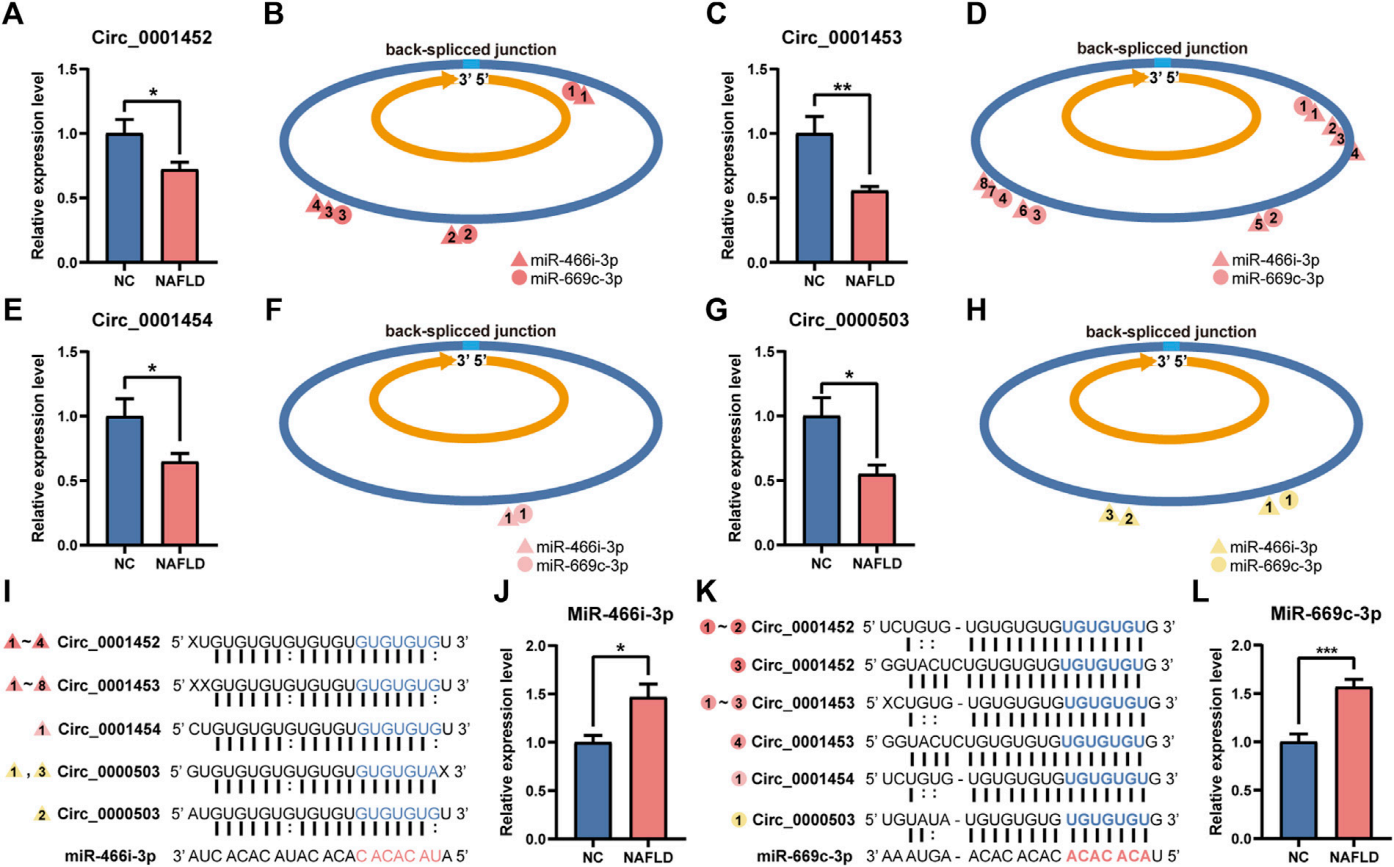
Deficiency of circ_0001452, circ_0001453, circ_0001454, and circ_0000503 activates miR-466i-3p and miR-669c-3p. **(A–H)** Hepatic circ_0001452 **(A)**, circ_0001453 **(C)**, circ_0001454 **(E)**, and circ_0000503 **(G)** exhibited expressive inhibition in the NAFLD group. They were generated by back-spliced junction with multiple miRNA response elements (MRE) specific to miR-466i-3p (triangle) and miR-669c-3p (circle) **(B,D,F,H)**. **(I–L)** miR-466i-3p **(I)** and miR-669c-3p **(K)** complemented to MREs of circ_0001452, circ_0001453, circ_0001454, and circ_0000503 by their ‘seed sequence’. Serial number of triangles and circles indicates the complementation sites of miR-466i-3p and miR-669c-3p, respectively. The miR-466i-3p **(J)** and miR-669c-3p levels **(L)** underwent significant upregulation after the loss of circ_0001452, circ_0001453, circ_0001454, and circ_0000503 in the NAFLD group. The data was presented as mean ± SEM. *, *p* < 0.05; **, *p* < 0.01; ***, *p* < 0.001.

In the NAFLD group, the hepatic levels of *LNCPINT*-derived circRNAs (circ_0001452, circ_0001453, circ_0001454) and circ_0000503 were lower compared to those in the NC group (Figures 5A,C,E,G). Deficient circRNAs generated by back-spliced junctions contained multiple MREs specific to miR-466i-3p and miR-669c-3p (Figures 5B,D,F,H). The complementary binding between the MREs of deficient circRNAs and the seed sequences of the corresponding miRNAs (Figures 5I,K) was abolished, leading to the reactivation of miR-466i-3p and miR-669c-3p. The hepatic expression level of miR-466i-3p and miR-669c-3p was also significantly upregulated in the NAFLD group compared to that in the NC group (Figures 5J,L).

### miR-466i-3p and miR-669c-3p inhibited the AMPK signaling pathway to promote the expression of lipogenic genes

**FIGURE 6:**
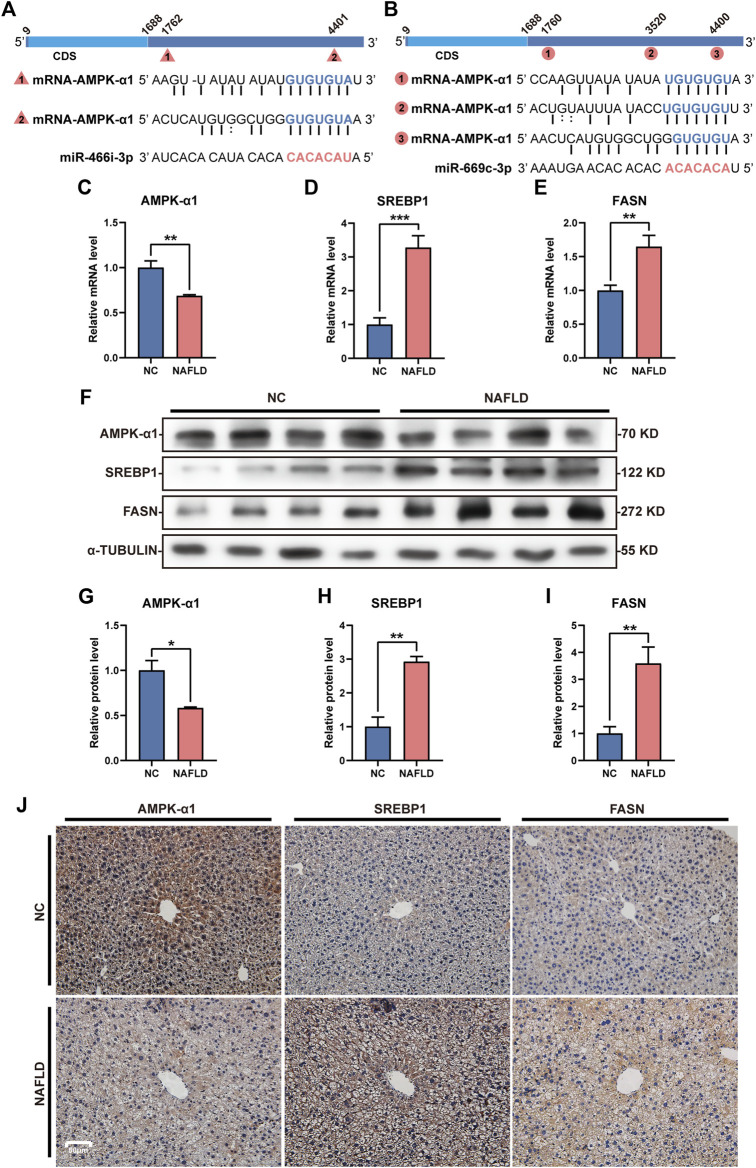
miR-466i-3p and miR-669c-3p activation induces AMPK-α1 downregulation and expressive promotion of lipogenic genes. **(A,B)** Both miR-466i-3p **(A)** and miR-669c-3p **(B)** targeted AMPK-α1 mRNA by the complementation of ‘seed sequence’ (miRNA) and 3′ untranslated region (mRNA). Triangles and circles labeling AMPK-α1 mRNA with serial numbers reflect the complementation sites of miR-466i-3p and miR-669c-3p, respectively. **(C–E)** miR-466i-3p and miR-669c-3p activation in the NAFLD group led to mRNA decrease in AMPK-α1 **(C)**, whereas transcriptional increase in SREBP1 **(D)** and FASN **(E)**. **(F–I)** Western blotting **(F)** semi-quantitatively confirmed the downregulation of AMPK-α1 **(G)** and upregulation of both SREBP1 **(H)** and FASN **(I)** in the NAFLD group. **(J)** Immunohistochemical staining revealed the expressive alterations of AMPK-α1, SREBP1, and FASN in hepatocytes. The data was presented as mean ± SEM. *, *p* < 0.05; **, *p* < 0.01; ***, *p* < 0.001.

Both miR-466i-3p and miR-669c-3p were recognized to repressively interact with AMPK-α1, one of the most important members of the AMPK family, through the seed sequence (located in the miRNAs) and the 3’ UTR (in the mRNA) (Figures 6A,B). The loss of *LNCPINT*-derived circRNAs in the NAFLD group lead to an increased level and functional activation of these miRNAs, and subsequent downregulation of AMPK-α1 expression at the transcriptional and translational levels (Figures 6C,F,G) in hepatocytes (Figure 6J). AMPK-α1 has been shown to inhibit the expression of downstream lipogenic genes, such as SREBP1 and FASN. Hence, as expected, miR-466i-3p- and miR-669c-3p-mediated AMPK-α1 downregulation markedly increased the mRNA and protein levels of both SREBP1 and FASN (Figures 6D–F,H,I). Immunohistochemical analysis revealed that hepatocytes with high-level of SREBP1 and FASN were more abundant in the NAFLD group than in the NC group (Figure 6J). Semi-quantitative analysis directly exhibited the expressive difference of AMPK-α1, SREBP1 and FASN between NC and NAFLD groups (Supplementary Figure S1). Finally, hepatic steatosis, the most characteristic pathological feature of NAFLD, also occurred in these hepatocytes (Figure 6J).

### Loss of LNCPINT-derived circRNAs and miR-466i-3p/miR-669c-3p activation is associated with hepatic TG concentration

**FIGURE 7:**
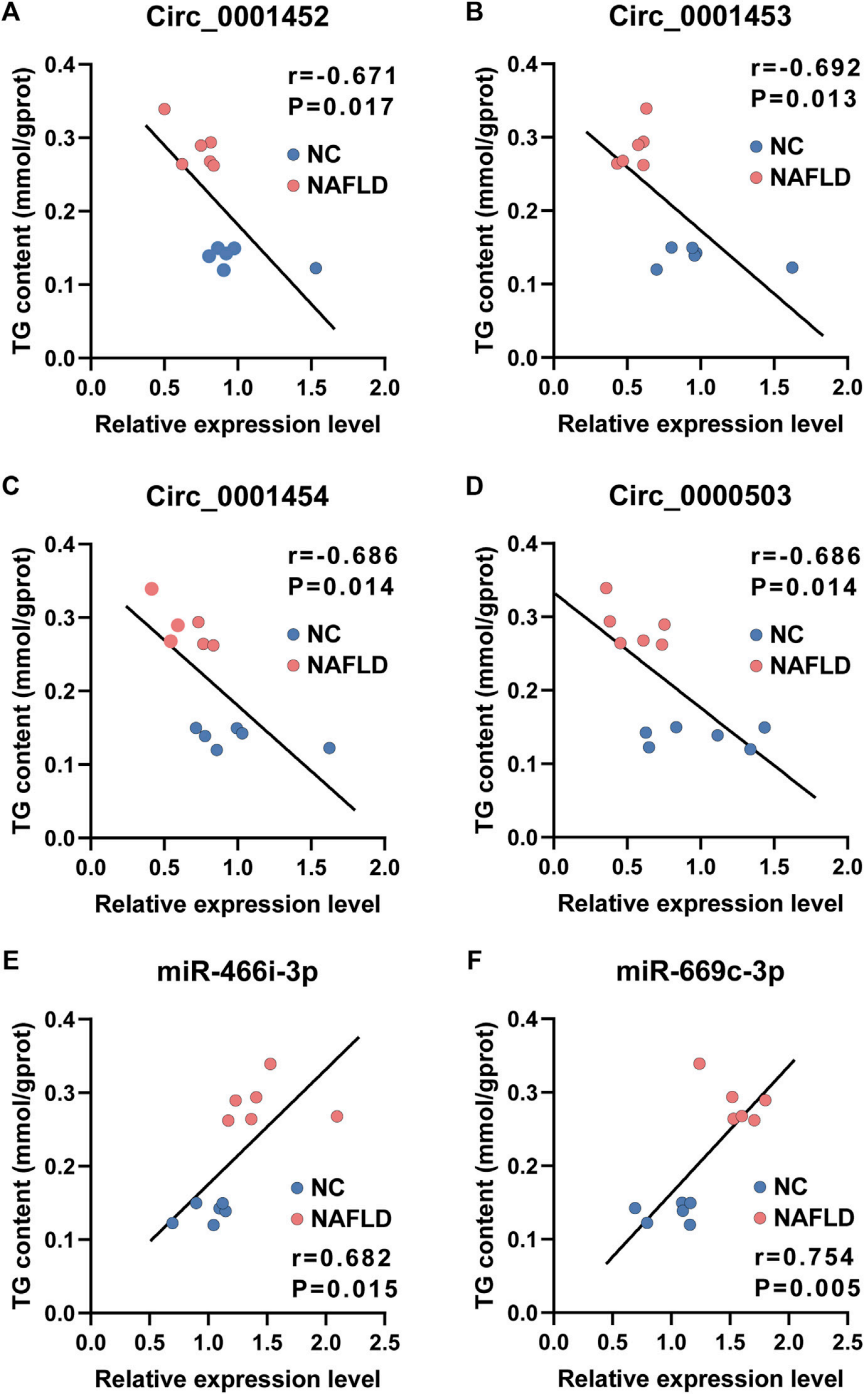
Loss of LNCPINT-derived circRNAs and miR-466i-3p/miR-669c-3p activation associated with hepatic TG concentration. **(A–D)** The levels of circ_0001452 **(A)**, circ_0001453 **(B)**, circ_0001454 **(C)**, and circ_0000503 **(D)** demonstrated significant association with hepatic TG content in a negative manner. **(E–F)** The expression level of both miR-466i-3p **(E)** and miR-669c-3p **(F)** correlated to the hepatic TG concentration with statistical significance.

Consistent with their putative roles in lipogenesis, the expression level of circ_0001452 (Figure 7A, r = −0.671, *p* = 0.017), circ_0001453 (Figure 7B, r = −0.692, *p* = 0.013), circ_0001454 (Figure 7C, r = −0.686, *p* = 0.014), and circ_0000503 (Figure 7D, r = −0.686, *p* = 0.014) was negatively associated with the TG concentration in the liver of rodents. Mice in the NAFLD group presented lower levels of *LNCPINT*-derived circRNAs and higher hepatic TG content compared with those of the NC group. The loss of *LNCPINT*-derived circRNAs lead to miR-466i-3p and miR-669c-3p activation, which was remarkably associated with the significant upregulation of hepatic TG levels (Figures 7E,F). In similar, both miR-466i-3p and miR-669c-3p levels were negatively correlated with the expression levels of *LNCPINT*-derived circRNAs, respectively (Supplementary Figures S2A,B). But the correlation was not observed between hepatic levels of both miRNAs and circ_0000503 (Supplementary Figures S2A,B).

## Discussion

CircRNAs, which used to be considered by-products of transcription, have now been accepted as versatile regulators of numerous physiological processes, including insulin secretion, neurogenesis, cartilage degradation among others (Hansen et al., 2013; Xu et al., 2015; Liu et al., 2016), as well as pathological abnormalities, such as diabetes, neurological disorders, cardiovascular diseases, chronic inflammatory diseases, and cancer, *etc.* (Holdt et al., 2016; Li et al., 2018a; Bai et al., 2018; Fang et al., 2018; Dong et al., 2019). To assess their involvement in NAFLD, their profile in the HFHC diet-induced NAFLD was subjected to transcriptome-wide sequencing with high fidelity. Interestingly, the expression profiles of NAFLD-specific circRNAs presented several characteristic features. First, circRNAs without circBase annotation dominated the circRNA profiles of both NC (24.63 ± 1.20%) and NAFLD (15.19 ± 4.21%) groups, showing a profile consistency with circRNA profiles of diabetic peripheral neuropathy, pulmonary fibrosis, and mouse brain (Lu et al., 2019; Zhang et al., 2020; Rahimi et al., 2021). Second, there were fewer circRNAs, with or without annotation, expressed in the NAFLD group than in the NC group. Third, the expression of NAFLD-related circRNAs underwent global downregulation. Among them, 28 showed a significant fold change and *p* value < 0.05. Taken together, these results suggested that the HFHC diet abrogated circRNA-dependent epigenetic regulation in NAFLD mice.

To further understand the mechanistic action of circRNAs in NAFLD, a circRNA-miRNA-mRNA regulatory network was established by assessing the complementary interaction of MREs, seed sequences, and 3’ UTR located in circRNAs, miRNAs, and mRNAs, respectively. Among the key circRNAs, circ_0001452, circ_0001453, and circ_0001454 were derived from *LNCPINT*, down-regulated in NAFLD mice, and targeted miR-466i-3p and miR-669c-3p. In the present study, increased levels and activity of miR-466i-3p and miR-669c-3p were observed upon the loss of LNCPINT-derived circRNAs. Palmitate exposure and metabolic oxidative stress have been shown to activate the expression of miR-466 and the miR-297-669 cluster (Li et al., 2011a; Druz et al., 2012), which further regulates NAFLD through peroxisome proliferator-activated receptor-gamma coactivator 1-α (PPARGC1A) (Mukushkina et al., 2020; Taghvaei et al., 2021). miR-669, which belongs to the same cluster, is also activated under the same conditions and is implicated in liver functions (Druz et al., 2012). Therefore, the activation of both miRNAs may promote lipogenesis, eventually leading to hepatic steatosis.

By binding to the seed sequence present in miRNAs, circRNAs inactivate miRNAs, thereby enhancing the transcription and/or translation of the target mRNAs. In contrast, circRNA loss reactivates miRNAs’ inhibitory effect over mRNAs (Hansen et al., 2013). Our experiments revealed many of the targets that miR-466i-3p and miR-669c-3p were shared. One of the common targets was AMPK-α1, which is crucial to the AMPK signaling pathway (Lei et al., 2019). KEGG analysis of miRNA targets confirmed they were highly enriched in the AMPK signaling pathway, which is intimately associated with NAFLD. The mRNA and protein expression levels of AMPK-α1 in the NAFLD group after circRNA-based reactivation of miR-466i-3p and miR-669c-3p were lower than in the NC group. Both miR-466i-3p and miR-669c-3p suppressed AMPK-α1 expression and regulated the AMPK signaling pathway in the liver.

The AMPK signaling pathway is known to play a central role in glycolipid metabolism (Li et al., 2011b; Wang et al., 2017; Lei et al., 2019; Zhu et al., 2019). Within the AMPK signaling pathway, AMPK catalyzes Ser372 phosphorylation of SREBP1 to prevent its cleavage-dependent activation and nuclear translocation (Li et al., 2011b). By regulating the transcription of lipogenic genes containing sterol regulatory element (SRE) motifs, such as FASN, acetyl-Coenzyme A carboxylase alpha (ACC1), and stearoyl-Coenzyme A desaturase 1 (SCD1), SREBP1 in the condition of phosphorylated inactivation reduces hepatocellular lipogenesis and triglyceride accumulation (Li et al., 2011b; Zhu et al., 2019). In contrast, inhibition of the AMPK signaling pathway by AMPK downregulation aggravates insulin resistance and hepatic steatosis under high-fat stimuli (Wang et al., 2017; Lei et al., 2019). In the NAFLD group, miR-466i-3p- and miR-669c-3p-related AMPK downregulation was accompanied by a significant increase in SREBP1 and FASN levels, which suggested that the activation of the AMPK signaling pathway was reduced. Altogether, these findings highlight that NAFLD with hepatic steatosis concomitantly occurred as a sequence of metabolic abnormality.

This study existed some limitations. The circRNA-miRNA and miRNA-mRNA interacting sites may be more than prediction, mainly due to the strict threshold in this study. Compared to the low-level threshold with score >140 and energy < −10 in miRanda prediction, our study employed a threshold with score >180 and energy < −30 for the highest accuracy and confidence in the prediction of circRNA-miRNA interactions (Li et al., 2018b; Li et al., 2019b; Li et al., 2020; Cao et al., 2021). Similarly, the miRNA-mRNA interactions identified by ≥ 6 databases and miRDB score ≥80 are supposed to be different, to some extent, from those obtained by one to three databases (Li et al., 2019b; Cao et al., 2021; Liu et al., 2021). Although the circRNA-miRNA and miRNA-mRNA interactions were of high confidence, *in vivo* and/or *in vitro* investigation will be valuable. Given the complicated regulation of AMPK signaling pathway in NAFLD, importance of the hub circRNAs and miRNAs filtered in this study needs further verification.

## Conclusion

**FIGURE 8:**
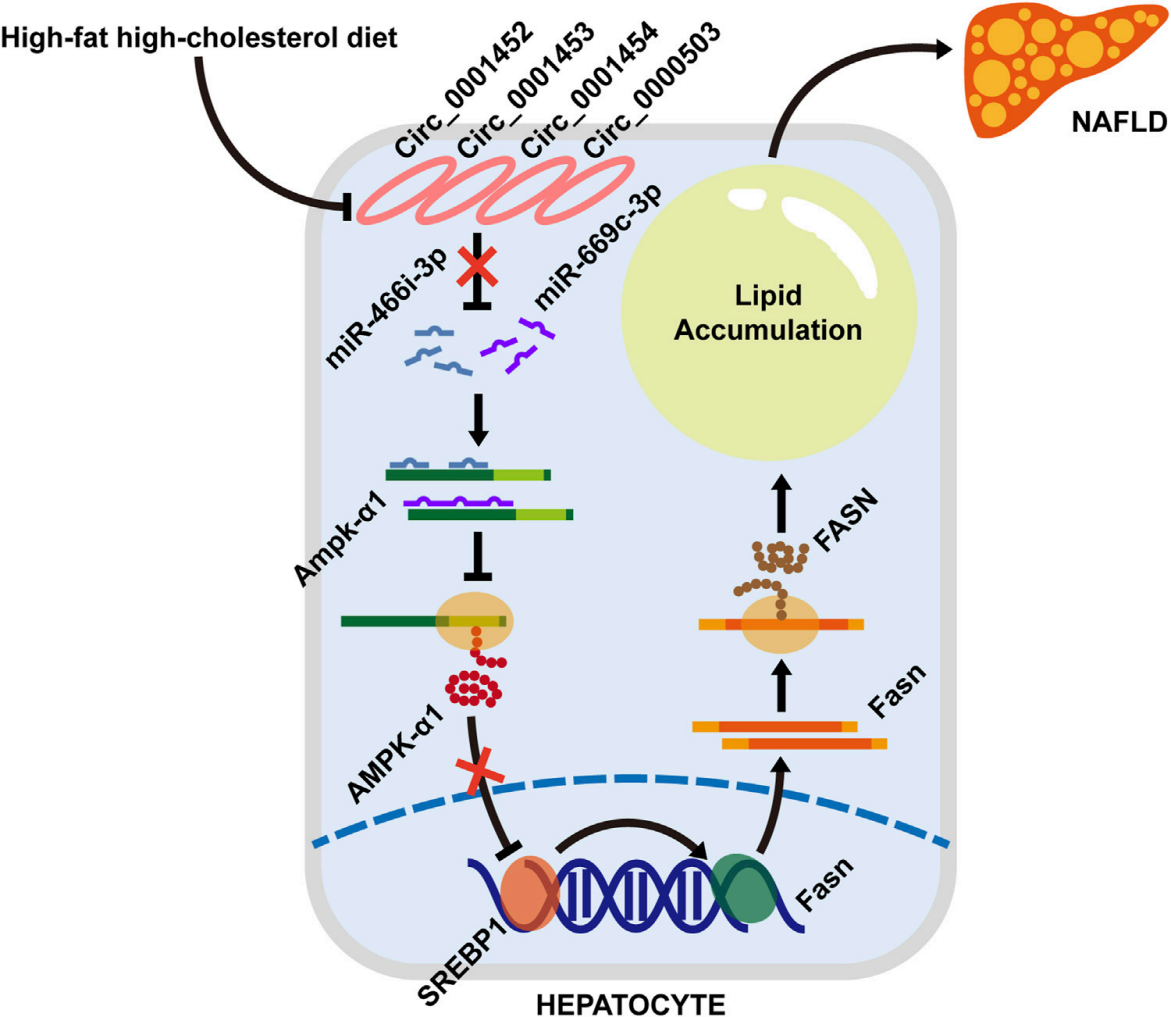
A schematic model depicting the loss of LNCPINT-derived circRNAs induces hepatocyte steatosis by miR-466i-3p- and miR-669c-3p-based inhibition of the AMPK signaling pathway. When compared to the normal diet (NC group), the HFHC diet (NAFLD group) leads to the expression loss of circ_0001452, circ_0001453, circ_0001454, and circ_0000503 in mouse hepatocytes. Most of these differentially expressed circRNAs derive from LNCPINT. The deficiency of LNCPINT-derived circRNAs abrogates their inhibitory effect on miR-466i-3p and miR-669c-3p, which further downregulate the AMPK-α1 expression via miRNA-mRNA complementation. Then AMPK-α1 downregulation reactivates the transcription and translation of lipogenic genes (SREBP1 and FASN). As result, excessive fatty acid synthesis and lipid accumulation give rise to the rodent NAFLD upon lacking LNCPINT-derived circRNAs.

Our results demonstrated that rodents with NAFLD fed with an HFHC diet present a dysregulated hepatic circRNA profile with 28 significantly downregulated circRNAs. Among these differentially expressed circRNAs, LNCPINT-derived circ_0001452, circ_0001453, and circ_0001454 were identified to play a pivotal role in the circRNA-miRNA-mRNA regulatory network. Their deficiency abrogated the circRNA-based inhibitory effect on both miR-466i-3p and miR-669c-3p, which further inactivated the AMPK signaling pathway via AMPK inhibition. Suppression of the AMPK signaling pathway, which leads to the transcriptional and translational promotion of lipogenic gene expression, may ultimately lead to hepatic steatosis (Figure 8).

## Data Availability

The datasets generated for this study can be found in the Sequence Read Archive (SRA) of NCBI with a BioProject ID: PRJNA810343 (http://www.ncbi.nlm.nih.gov/bioproject/810343).
