# Supplementary material for: Profile analysis and functional modeling identify circular RNAs in nonalcoholic fatty liver disease as regulators of hepatic lipid metabolism
Source: Front Genet. 2022 Sep 15;13:884037. doi: 10.3389/fgene.2022.884037 (PMC9520628; doi:10.3389/fgene.2022.884037)
Supplement: Supplementary file 1 [file DataSheet1.docx]

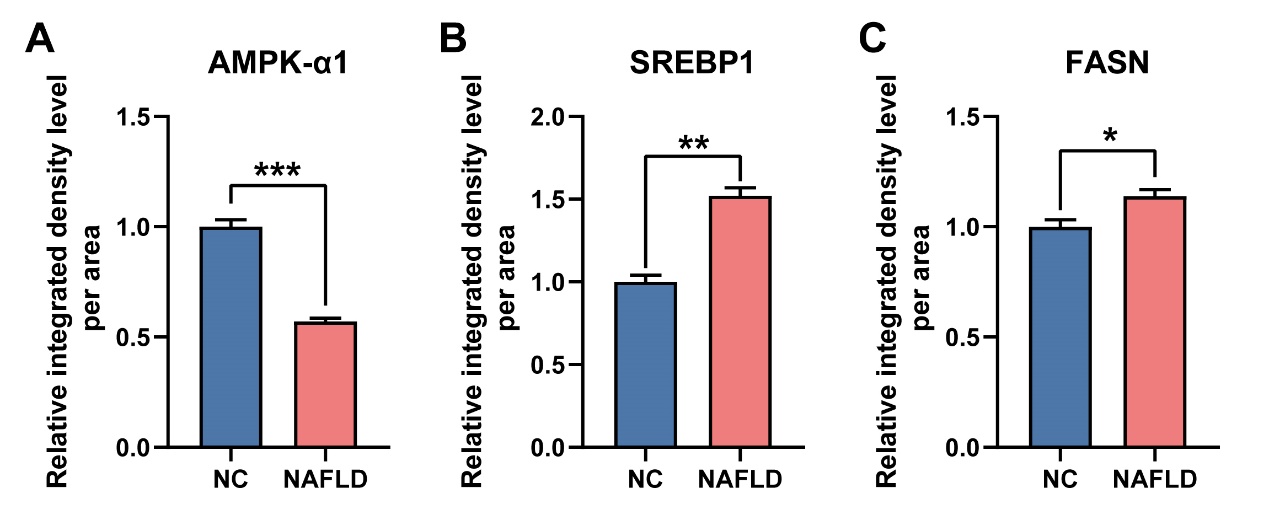


FigureS1. Semiquantitative analysis of immunohistochemical signals. The integrated density adjusted by analyzed area showed the significant downregulation of AMPK-α1 (A), and then upregulation of SREBP1 (B) and FASN (C), in the NAFLD group.


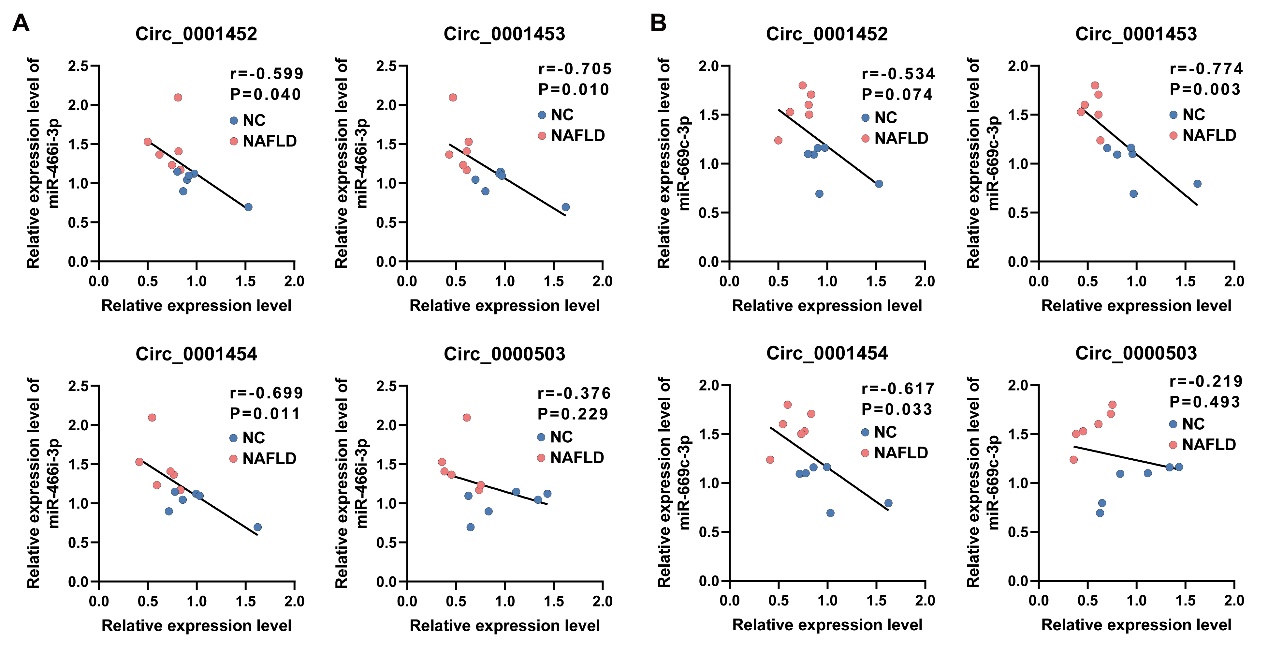


FigureS2. Correlation between expression levels of *LNCPINT*-derived circRNAs and miRNAs. (A) The association between circRNAs and miR-466i-3p, of which each *LNCPINT*-derived circRNA reached the significant level. (B) The association between circRNAs and miR-669c-3p, of which circ_0001453 and circ_0001454 reached the significant level.
